# Current and Future Demand for Health Services Researchers: Perspectives from Diverse Research Organizations

**DOI:** 10.1111/1475-6773.12999

**Published:** 2018-06-12

**Authors:** Eugene Rich, Anna Collins

**Affiliations:** ^1^ Mathematica Policy Research Washington DC

**Keywords:** Qualitative research, health workforce, distribution, incomes, training, business and management

## Abstract

**Objective:**

The Affordable Care Act and Medicare Access and CHIP Reauthorization Act are changing access to, and delivery of, health care in the United States, with potential implications for the field of health services research (HSR). We therefore investigated employers' perceptions of demand for individuals to conduct HSR, the competencies required for success, and implications for HSR training programs.

**Data Source:**

Interviews conducted in August 2016 with 21 key informants at a range of U.S.‐based HSR organizations.

**Study Design:**

We conducted a semistructured, qualitative, telephone interview study to explore relevant topics.

**Data Collection/Extraction Methods:**

Interviews with respondents were transcribed from recordings and then synthesized by respondent organization type and topic area.

**Principal Findings:**

Most respondents reported recently hiring health services researchers, and most anticipated hiring additional such researchers in the future. Most respondents emphasized the abilities to analyze data, work in teams, and engage with stakeholders. Finally, most respondents recommended that potential recruits gain *real‐world* experience during their academic training.

**Conclusions:**

Our interviews indicated that current and future demand for health services researchers is strong. They also suggested that, as a field, HSR will continue to draw together individuals from a variety of backgrounds to inform a diverse array of decision makers.

AcademyHealth defines health services research (HSR) as “the multidisciplinary field of scientific investigation that studies how social factors, financing systems, organizational structures and processes, health technologies, and personal behaviors affect access to healthcare, the quality and cost of healthcare, and ultimately, our health and well‐being.” There is an ongoing interest in the training and development of scholars in this field (Forrest et al. 2009; Pittman and Holve 2009), especially as of late. In recent years, health care in the United States has begun to undergo major changes in both access and delivery, driven by the passage of the Affordable Care Act (ACA) in 2010 and the Medicare Access and CHIP Reauthorization Act (MACRA) in 2015 (Rosenbaum 2011; Blumenthal, Abrams, and Nuzum 2015; Antos and Capretta 2017; Fryhofer et al. 2017). The public and private initiatives undertaken in response to these policy changes engage a broad range of the topics relevant to HSR, and thus could substantially influence current and future demand for health services researchers (HSRs).

Accordingly, in 2016, we undertook to investigate the perceived need for HSRs, as part of a larger effort—supported by AcademyHealth and the Agency for Healthcare Research and Quality (AHRQ)—to understand the current HSR environment and training programs pertinent to the field. We engaged leaders at a range of U.S.‐based organizations that conduct HSR to explore their current and future need for HSRs, as well as their perceptions of the requisite skills and experiences at types of organizations like theirs. This work is intended to inform HSR practitioners, employers, scholars, and educators across the field to ensure diverse HSR organizations have access to researchers with the competencies critical to providing informative and impactful research.

## Methods

The results presented in this paper derive from interviews with representatives from a range of U.S.‐based organizations that conduct HSR. To obtain a representative sample of such organizations and key informants, we consulted with leaders of the HSR workforce task force overseeing an AcademyHealth initiative “to understand the current health services research workforce and maximize its future.” Based on task force input, we recruited interviewees from among relevant HSR program leaders on the AcademyHealth Board, AcademyHealth Corporate Council,^1^ and AcademyHealth Methods Council.^2^ Ultimately, 21 of our 22 invitees participated in this study. All are individuals with substantive HSR experience who held leadership roles within their respective organizations at the time the interviews occurred; the organizations themselves were classified into six categories: health care delivery systems (number of respondents [*n*] = 5); life sciences companies (*n* = 4); health information technology and data analytics companies (*n* = 3); payers and purchasers (*n* = 3); university‐based research programs (*n* = 3); and nonpartisan U.S. policy research organizations (*n* = 3) (U.S. Health Policy Gateway). We conducted semistructured, 30‐minute telephone interviews with each respondent individually.

As noted above, the motivation for this study was an AcademyHealth initiative addressing issues relevant to the HSR workforce. The task force leading this initiative identified the five broad topics presented below to inform this aspect of its work. Our semistructured interview protocol was thus organized around and designed to address these five topics. For each of these topics, we developed related open‐ended questions to guide our interviews; the number of questions developed for each topic and ultimately placed in our interview protocol is noted in parentheses below. As with the five broader topics, the development of these individual open‐ended questions was likewise informed by guidance received from the aforementioned AcademyHealth task force.

1. Current and future demand for HSRs (two questions in our interview protocol);
2. The adequacy of the current supply of HSRs (one question in our interview protocol);
3. Critical competencies for success in the field of HSR (five questions in our interview protocol);
4. The nature of both the work assigned to HSRs and their role in procuring financial support for that work (three questions in our interview protocol); and
5. The changes HSR training and development programs should consider undertaking to meet future employer needs (two questions in our interview protocol).

To guide the portion of the interview addressing critical competencies for success in HSR, we also drew upon three reports summarizing knowledge and skills specific to HSR: Forrest et al. (2009); Geonnotti, Rich, and Esposito (2014); and Pittman and Holve (2009). Based on these reports, we developed a *pick list* of competencies relevant to three broad domains: knowledge; methodological and analytic skills; and general research skills. This list (Table 1) was provided to respondents in advance of their interviews, and was used as a starting point for respondents during the interviews when they were asked to identify what they consider the priority competencies for success in HSR. The interview protocol was ultimately reviewed and approved by AcademyHealth project leads, and then pilot tested with two HSR organization leaders experienced in researcher recruitment. As needed, the protocol was revised based on these leaders' feedback.

**Table 1:** HSR Competencies

Specific areas of knowledge	Knowledge…
	… from specific intellectual disciplines (both theoretical and conceptual)^a^,^b^Examples: clinical informatics, public policy, political science, economics, epidemiology, management sciences, psychology, sociology, statistics, health systems engineering
	… from clinical disciplines^a^,^b^
	… of specific aspects of the health care system^a^Examples: the structures, performance, quality, policy, and/or environmental context of health and health care
	… of sources of primary health and health care data for health services research^a^,^b^,^c^
	… of public and private sources of secondary data for health services research^a^,^b^,^c^
Specific methodological and analytic skills	The ability to…
	… formulate solutions to health policy problems^a^
	… pose innovative and important health services research questions, informed by stakeholder needs^a^,^c^
	… conduct systematic reviews of the literature^a^,^c^
	… develop and apply relevant theoretical and conceptual models^a^,^c^
	… select appropriate study designs^a^,^c^
	… obtain primary health and health care data^a^
	… assemble secondary data from existing public and private sources^a^
	… use conceptual models to specify study constructs for a health services research question^a^
	… develop variables that reliably and validly measure specific study constructs^a^
	… implement research protocols that ensure reproducibility of the science^a^,^b^
	… use appropriate analytical methods^a^,^b^,^c^
	… analyze large datasets, including EHR information^c^
General research skills	The ability to…
	… work collaboratively in multidisciplinary teams^a^,^b^
	… effectively communicate findings and implications^a^,^b^
	… write proposals^a^
	… translate health services research into policy and practice^a^
	… ensure the ethical and responsible conduct of research^a^
	… collaborate with stakeholders^a^,^c^

a Forrest et al. (2009).

b Pittman and Holve (2009).

c Geonnotti, Rich, and Esposito (2014).

We framed the meaning of HSR for our interviewees as follows: “I want to take a moment to note how we are defining health services research in the context of this study.” We then referred to the AcademyHealth definition of HSR given above, which was also emailed to each respondent the day prior to his/her interview. If asked for clarification regarding the expected qualifications or roles of the employees in question, we indicated that we were inquiring about those individuals who “lead HSR projects or tasks at your organization.”

At the beginning of each interview, verbal consent was obtained to record the interview with the assurance of strict anonymity. Present at each interview was an analyst who took careful notes during the interview; immediately after the interview ended, the analyst listened to and transcribed the interview recording as a means of enhancing the notes taken while the interview was in progress. Each transcript was then reviewed by the interviewer—a senior researcher—to ensure accuracy and completeness. After all the transcripts were produced and reviewed, the responses were transferred by an analyst to a table shell arranged by organization type (the table columns) and interview question (the table rows). The interview questions (table rows) were further organized by the five topics listed above using header rows. In the end, all the cells in the table presented our interviewees' verbatim responses to each protocol question by organization type. The fully populated table shell was reviewed by the interviewer to ensure that the content from each interview had been placed in the table appropriately.

Our analysis of our interviewees' responses followed the directed content analysis approach described by Hsieh and Shannon (2005), through which a researcher explores a specific subject area (in this case, current and future demand for HSRs) by focusing on prescribed key concepts relevant to the subject area (in this case, the five topics listed above), including developing interview questions based on these key concepts. In this study, we began with a set of five topics identified by the aforementioned AcademyHealth task force; we subsequently developed an interview protocol based on these five topics—again utilizing input from content experts, including leaders of the AcademyHealth task force—and in the final stage, extracted key takeaways related to each of these topics. With regard to this final stage, a researcher and analyst extracted, in collaboration, key takeaways from each cell in the aforementioned analysis table. This proceeded through several steps:

1. Within each cell of the analysis table, the analyst reviewed all the interviewees' responses, highlighting similar responses or similar pieces of responses in the same color;
2. Once highlighting was complete, the analyst rereviewed the content of each cell and based on the highlighting scheme, which showed which responses to each question emerged most frequently among members of a given organization type, added 1–3 “key takeaway” sentences at the bottom of each cell; and
3. The researcher independently reviewed all the cells in the fully populated analysis table and confirmed whether he agreed with both the highlighting and the summary line or lines at the bottom of each cell. If there were discrepancies in interpretation for a given cell between the analyst and researcher, the two would independently reevaluate the complete interview transcripts for that question and organization type to come to an agreement about the key takeaway message(s).

The key takeaways within each cell, when finalized, were then transferred to an early draft of this report; as needed, the full analysis table was referenced during the report writing process for direct quotes or supporting details.

In the remainder of this report, we primarily present the ideas and thoughts expressed in reaction to a given interview question that reflect the majority opinion among interviewees from a certain organization type; if an idea presented below was expressed by less than a majority of interviewees within a given organization category (i.e., by fewer than three interviewees among organization types where *n* = 4 or 5, or by fewer than two interviewees where *n* = 3), we make this clear in the text. In some places, we also provide illustrative or supporting quotes directly from the interviews if we determined they would help to convey sentiments expressed by a majority of respondents.

In the next section (*Findings*), we discuss our key takeaways from the interviews by both topic area and organization type: health care delivery systems; life sciences companies; health information technology and data analytics companies (hereafter referred to as *data analytics*); payers and purchasers (hereafter referred to as *payers*); university‐based research programs (hereafter referred to as *academics*); and nonpartisan U.S. policy research organizations.

## Findings

### Current and Future Demand for HSRs

Twenty of the 21 total respondents indicated that their organizations had hired additional HSRs within the previous two years. Furthermore, fifteen of the 21 respondents anticipated that their organizations will hire HSRs over the next three years. Respondents from delivery systems predicted that their organizations' need for HSRs will increase over the next three years given changing approaches to payment for care, and growing focus on both value over volume and population health. Respondents from data analytics held similar views; most anticipated increased demand for HSR‐related services among their clients over the coming years. Those from policy research organizations also anticipated a growing need for HSRs in the next several years. They cited several reasons for this, including additional work related to the implementation of recent legislation, especially the need for analyses of new payment models and delivery system reform initiatives, including the aforementioned MACRA, as well as similar initiatives being undertaken by private payers.

Academics were more reserved in predicting need for additional faculty to conduct HSR in the near future. All noted that demand will be influenced by the availability of funding. Some also noted the changing health care and delivery system environments, which might create new opportunities for university‐based HSRs at their respective institutions.

Among those respondents from both payers and the life sciences, predictions of future need were mixed. Some anticipated no additional hiring over the next few years, some that future need will remain consistent with past need, and some an increased need for HSR project leaders.

### The Adequacy of the Current Supply of HSRs

Most respondents perceived the current pool of HSR candidates as sufficient in terms of both numbers and quality. Many respondents, however, indicated that finding candidates with specific, high‐priority skills can prove challenging—a topic explored in depth in the subsequent section (*Critical competencies for success in the field of HSR*). For instance, policy research respondents noted the challenge in finding candidates with client communication and policy writing skills. Data analytics, life sciences, payer, and delivery system respondents all noted difficulty in finding candidates with a “business mindset” (e.g., candidates able to understand the practical realities inherent to success in the organization's line of business) or relevant past work experience.

### Critical Competencies for Success in the Field of HSR

In Table 2, we present the competencies respondents cited most frequently in response to the following interview questions: “Thinking about your organization's current recruitment efforts, what do you consider the top three most critical [(1) areas of knowledge; (2) methodologic and analytic skills; or (3) general research skills] for recruits to demonstrate?” In answering these questions, respondents were asked to consider the specific competencies included in Table 1, but also invited to note any priority competencies not shown there.

**Table 2:** Most Frequently Cited Key Competencies for Success in HSR

	Delivery System Respondents (*n* = 5)	Life Science Organization Respondents (*n* = 4)	Data Analytics Respondents (*n* = 3)	Payer Respondents (*n* = 3)	Academics Respondents (*n* = 3)	Policy Research Organization Respondents (*n* = 3)
Areas of knowledge	Health care delivery systems Public policy Economics Epidemiology Health systems engineering Clinical informatics	Public policy Economics Epidemiology Health systems engineering Utilization and patient outcomes	Statistics Social sciences Clinical informatics Computer science Operations research Utilization and patient outcomes Health communications	Public policy Economics Statistics Epidemiology Health systems engineering Management sciences	Health care delivery systems Public health Economics Statistics Epidemiology Health systems engineering Clinical informatics Clinical disciplines	Health care delivery systems Economics Social sciences
Methodological & analytic skills	The abilities to: Pose important research questions^a^ Use conceptual models to specify study constructs Develop and apply relevant theoretical and conceptual models Analyze large datasets^a^	The abilities to: Pose important research questions^a^ Select appropriate study designs Use appropriate analytical methods Analyze large datasets^a^ Formulate solutions to health policy problems	The abilities to: Pose important research questions^a^ Develop and apply relevant theoretical and conceptual models Analyze large datasets^a^	The abilities to: Pose important research questions^a^ Conduct systematic reviews; Analyze large datasets^a^ Formulate solutions to health policy problems	The abilities to: Pose important research questions^a^ Select appropriate study designs Use appropriate analytical methods Develop and apply relevant theoretical and conceptual models Analyze large datasets^a^	The abilities to: Pose important research questions^a^ Select appropriate study designs Analyze large datasets^a^ Formulate solutions to health policy problems
General research skills^b^	Across all organization types, respondents emphasized the abilities to: Work collaboratively in multidisciplinary teams and with stakeholders Translate HSR into policy and practice Effectively communicate findings and implications					

a These abilities were consistently cited as high priority by respondents across all organization types.

b To simplify this aspect of the interview, we acknowledged at the outset that all HSR organizations desire individuals who can “ensure the ethical and responsible conduct of research.”

In considering critical areas of knowledge, several delivery system and life sciences respondents—in addition to noting some of the areas presented in Table 1—also emphasized the importance of securing *generalists*. One delivery system respondent described these as: “The kinds of people [who] are not necessarily experts in a particular area. They're more [individuals] who can apply their knowledge base and make connections across different sectors.”

Regarding methodological and analytic skills, respondents across all six organization types cited the ability to analyze large datasets, including electronic health record (EHR) information. In addition to the competencies cited in Table 1, some respondents also emphasized the importance of familiarity with *mixed methods*. As one delivery system respondent explained, capturing a sentiment expressed by respondents across organization types, “It's impossible to do an evaluation of a complex intervention in a delivery system setting without using mixed methods—both quantitative and qualitative training is therefore crucial.”

In addition, respondents almost universally valued the abilities to understand the perspectives of, and effectively collaborate with, stakeholders, although the definition of *stakeholder* appeared to vary by organization type. Delivery system respondents emphasized the importance of being able to engage with *patients and consumers* in particular. Data analytics respondents, on the other hand, noted the importance of being able to collaborate with *clients*, and to understand the data needs of those clients' unique businesses. Respondents in policy research noted the importance of being able to collaborate and communicate effectively with *policy makers*.

We also asked respondents whether they would be prioritizing different skills in their future recruitment of HSRs. The ability to analyze large datasets, including EHRs, was consistently noted as an emerging priority by academic, life sciences, and policy research respondents. This skill was noted as an existing priority among delivery system and data analytics respondents, although one data analytics respondent noted a growing need for researchers skilled in “machine learning” specifically. Delivery system respondents also noted a growing emphasis on the ability of HSRs to conduct implementation research work, such as helping to implement and assess delivery system improvements in response to new payment models.

### Nature of the Work Assigned to HSRs

We also asked our respondents about the nature of the work performed by HSRs at their respective organizations, as well as their role in securing financial support for that work. In Table 3, we present our findings related to these topics. Based on the responses we received, it appears that HSRs working with payers or in the life sciences are not typically expected to generate extramural support for their work, but instead to focus on projects and tasks related to the needs of parent organizations. Interestingly, among the delivery systems respondents we interviewed, they also indicated that HSRs at their units were not typically focused on procuring funding, noting that much of their work is internally financed by the delivery organization; two respondents did note that their organizations occasionally pursue supplementary external support.

**Table 3:** Nature of the Work Performed by HSRs

	Delivery System Respondents (*n* = 5)	Life Science Organization Respondents (*n* = 4)	Data Analytics Respondents (*n* = 3)	Payer Respondents (*n* = 3)	Academics Respondents (*n* = 3)	Policy Research Organization Respondents (*n* = 3)
Types of projects and tasks led by HSRs	Conduct clinical trials Conduct and/or evaluate delivery system interventions Patient and utilization data analyses	Research project design and oversight Conduct clinical trials Data analyses (diverse sources) Synthesizing and disseminating results	Address unique data collection and analysis needs of clients	Interventions and evaluations Literature reviews Surveys Patient utilization and financial data analyses	Policy research project design and execution Data collection (qualitative and quantitative) Data analyses (diverse sources) Synthesizing and disseminating results	Policy research project design and execution Data collection (qualitative and quantitative) Data analyses (diverse sources) Synthesizing and disseminating results
Sources of funding for HSR work (internal vs. external)	Internal	Internal	External	Internal	External	External
The role of HSRs in procuring funding	Limited role since most funding is supplied internally	Limited role since most funding is supplied internally	HSRs generally work as part of teams to develop responses to “requests for proposals.”	Limited role since most funding is supplied internally	HSRs are generally expected to “support their own work” by leading or independently executing proposals.	HSRs generally work as part of teams to develop responses to “requests for proposals.”

Of course, the type of intramural HSR work funded by these different types of organizations varied considerably. At delivery systems, projects might range from evaluations of new delivery system interventions to identification of high‐risk populations. Multidisciplinary teams are also key to the work. As one respondent noted, “We have clinical folks that help to design interventions, and HSRs that help to design implementations … and then conduct the evaluations.” At life sciences companies, HSR projects might focus not only on the costs and benefits of new products (such as drugs or devices), but also on addressing the concerns of external stakeholders, including policy makers and consumers. Projects conducted by payers might include formal evaluations of internally developed payment reforms and quality improvement initiatives, or they may address HSR questions or policy options of interest to specific stakeholder partners.

At policy research organizations, HSR employees work almost exclusively on research contracts funded by external clients, with efforts ranging from prospective evaluations of policy changes, to observational studies, to systematic reviews of available evidence, to technical assistance for policy makers or other stakeholders influenced by new public policies. Like their colleagues at delivery systems, HSRs at policy research organizations are typically not individually responsible for securing their own project funding. Instead, they work in teams to prepare proposals and conduct work. The process for securing support for HSR at data analytics organizations is similar, with investigators working on tasks funded by contracts with diverse clients needing expertise analyzing health information technology (HIT), clinical, or payer‐derived data to support decision making. Multidisciplinary teams are a feature of the HSR work in these organizations as well.

Academic respondents also acknowledged that multidisciplinary teams are critical. However, university‐based investigators were described as more individually and independently responsible for securing external financial support for their projects. As one respondent observed, “Anybody on the tenure track has to have a certain amount of PI [principal investigator] activities for promotion.” At both policy research organizations and academic institutions, ongoing intramural support for individual research projects and programs is typically not provided.

### Recommended Changes to Existing HSR Training Programs to Meet Future Needs

We ended the interviews by asking respondents whether they had any recommendations for advanced academic or HSR training programs, particularly in terms of how they can help prepare individuals to conduct HSR at organizations like theirs. The vast majority of respondents emphasized the need for some type of *real‐world* HSR experiences during training. Naturally, delivery system respondents saw potential value in formal HSR training programs offering their students rotations through integrated delivery systems. The majority of the delivery system respondents agreed that advanced academic training programs should in general find ways to cultivate individuals' “practical” skills, such as skills in translation and rapid cycle evaluation, as well as their workforce‐applicable “leadership” skills, such as the abilities to work with multidisciplinary teams and clinicians. Policy research respondents, too, expressed a desire for training programs to prepare individuals for project leadership positions.

Academics likewise expressed the view that trainees should be given more opportunities to engage with stakeholders, clinicians, community partners, and patients, as well as to participate in multidisciplinary research teams. In terms of the specific subjects academics would like to see receive more emphasis in training programs, they mentioned a need for more instruction in causal inference, informatics and the analysis of EHR data, and multimethods research.

Life sciences, payer, and policy research respondents also indicated that trainees would benefit from opportunities to complete rotations or internships in organizations such as theirs. Policy research respondents in particular emphasized a need for enhanced training in “policy writing” and research methods, especially mixed methods, while data analytics respondents hoped to see more stress on computation skills, as well as the ability to understand health care financing data.

## Discussion

Our interviews indicate that there is both substantial demand currently for HSRs, as well as strongly anticipated future demand. Expectations of near‐term growth in demand seem primarily driven by the need for individuals who can use the tools of HSR to inform and/or evaluate changing modes of care delivery and payment. Recent federal policies such as the MACRA, as well as innovations by private payers, were noted as important contributors to this demand.

Academics in particular noted that their future need for HSRs would continue to be influenced by the availability of federal research funding. Grant support for HSR from federal agencies has been flat, or in some cases declining, over the past five years (Cavarocchi, Ruscio, Dennis Associates 2017). Since the time of our 2016 interviews, grant funding for HSR has not become more certain. Indeed, current administration proposals to restructure overall research spending, and to merge AHRQ into the NIH, have raised additional concerns (Katz and Wright 2017). While some commentators have suggested an appropriately structured integration of AHRQ into the NIH might be beneficial for the field (Bindman 2017), such uncertainty can affect career choices. Our interviews conducted prior to these most recent proposals suggest that, at least for HSR, the unpredictability of grant funding may not have been causing young HSR‐trained scholars to leave the field, but rather to find employment in other types of organizations or academic units. It appears that in many of these organizations, support for HSR may not rely on federal research grants, but rather on contracts with government agencies or industry clients, or alternatively, funding from payer, life sciences, or delivery organizations themselves. The continued interest of the current administration in developing and implementing new modes of provider payment to achieve Health & Human Services (HHS) goals suggests these motivations to undertake HSR relevant to delivery system reform will likely continue unabated (U.S. Department of Health & Human Services 2017).

These continued HHS priorities, combined with diverse interviewee emphasis on delivery system‐related HSR work, may have implications for the competencies demanded of upcoming HSRs. For example, many respondents noted that knowledge of health care delivery systems is essential to success among their recruits. Of course, the emphasis on delivery system reform also has implications relevant to demand for specific methodologic skills. One example is the prominence of the need for skills in *mixed methods* to evaluate complex delivery system interventions. The growth in both the amount and sophistication of data developed during health care operations, including EHR information, was also noted as having implications for the methodological skills demanded of future HSRs. Most respondents noted the importance of skills in the analysis of large datasets, while many also noted that experiences working with EHR data specifically are particularly important, though in short supply.

Essentially, all respondents emphasized the importance of skills relevant to understanding the perspectives of *real‐world* stakeholders. Of course, this may reflect the fact that the large majority of the organizations represented by our respondents (18 of 21) focus on addressing the needs of such stakeholders, rather than the community of HSR scholars reflected among peer reviewers of research grant applications. Not surprisingly, most respondents also emphasized the importance of gaining *real‐world experience* as part of HSR training programs. These respondents encouraged program directors to provide substantive experiences in the types of settings where many HSRs may be working in the future, with an eye toward showing trainees how data are being used to drive decisions on policy and care delivery.

The methodology behind this study presents inevitable limitations. We interviewed only 21 individuals stratified across six organization types. This means that only three to five representatives from each type of organization were interviewed. Moreover, to accommodate the time constraints of our busy interviewees, each interview was scheduled for a maximum of 30 minutes, which sometimes limited opportunities for follow‐up and clarification. As noted previously, our interviews focused only on the five topics presented above, which may not encompass all of the factors relevant to the recruitment and employment of individuals trained in HSR. Finally, these interviews occurred in the summer of 2016, following passage of key legislation like the ACA and MACRA, but prior to more recent changes in federal initiatives relevant to health care and HSR.

Our small sample size also means we cannot inform the important question of “where the jobs will be” for future HSR program trainees. Although five respondents were leaders of HSR programs in organized delivery systems, representatives from among the other organization types described substantive partnerships with health care systems. Thus, we cannot ascertain the degree to which the dominant future employer for HSR training program graduates will be health care systems themselves or other organizational partners. Nor can we know if most future health care system‐based units will include a substantial focus on extramurally funded work, such as that of some long‐standing members of the Health Systems Research Network, or if the more internally focused work environment reflected by our respondents is required to efficiently and effectively meet internal delivery organization priorities. However, we did hear consistently that during this time of health care system transformation, new HSRs must understand these care delivery settings, and be able to communicate effectively with clinicians and clinical program leaders in order to generate actionable evidence.

Although our respondents were clear about their demand for HSRs, our findings also suggest that no single academic discipline addresses the entirety of the diverse set of competencies and perspectives required of HSR practitioners. At the same time, HSR omnicompetence is neither expected nor required. As evidence of this, all respondents affirmed that HSR at their organizations is conducted in multidisciplinary teams. In addition, most use a collaborative approach to securing financial support for their HSR projects.

As a result, the field of HSR is likely to continue to draw together scholars with a wide variety of formal training backgrounds and methodological skills. For many HSRs, the applied nature of their work will mean an emphasis on understanding the needs of the specific decision makers they are seeking to inform, and the distinct settings in which these decisions occur. What unifies these disparate scholars of HSR is the focus on how to use a breadth of content knowledge and variety of research skills to build evidence that promotes the health and well‐being of the public. As one respondent succinctly concluded, “We're looking for people with basic [HSR] research knowledge, data analytics skills, study design skills, and a humanistic side.”

## Supporting information

Appendix SA1: Author Matrix.

## Notes

1 The purpose of the Corporate Council is to better connect AcademyHealth to the key actors in the health care industry who are seeking or using HSR, and increasingly producing HSR. The Council will “inform the planning, development, implementation, and evaluation of new member benefits, programs, and services which are responsive to the research and evidence needs of the range of industry sectors represented by Council members.” (http://www.academyhealth.org/node/303)

2 The Methods Council has four primary responsibilities: (1) to assist AcademyHealth in developing strategies for professional development in HSR; (2) to provide guidance to the Annual Research Meeting planning committee on emerging and relevant methods themes; (3) to provide guidance and relevant content for HSRMethods.org; and (4) to provide guidance on ongoing AcademyHealth projects and activities on an ad hoc basis. (http://www.academyhealth.org/node/302)
